# Protective and Pathogenic Antibodies to Citrullinated Antigens

**DOI:** 10.1111/imr.70165

**Published:** 2026-08-26

**Authors:** Outi Sareila, Yibo He, Zhongwei Xu, Changrong Ge, Erik Lönnblom, Rikard Holmdahl

**Affiliations:** ^1^ Department of Medical Biochemistry and Biophysics, Medical Inflammation Research Karolinska Institutet Stockholm Sweden; ^2^ Department of Medical Biochemistry and Microbiology, Medical Inflammation Research Uppsala University Uppsala Sweden; ^3^ National‐Local Joint Engineering Research Center of Biodiagnosis and Biotherapy The Second Affiliated Hospital of Xi'an Jiaotong University Xi'an Shaanxi China

**Keywords:** anti‐citrullinated protein antibodies, autoantibodies, B cells, diagnostics, pathogenic mechanisms, protective immunity, rheumatoid arthritis

## Abstract

Autoimmune diseases start years before clinical onset. In rheumatoid arthritis (RA), autoantibodies to citrullinated proteins (ACPA) and to modified IgG (rheumatoid factors, RF) can be detected many years before the inflammatory attack on cartilaginous joints. It has been assumed that these antibodies are pathogenic. We show here that many of the antibodies produced before disease onset are protective although there are likely also pathogenic clones directed to cartilaginous joints. To determine the function of these antibodies, it is crucial to analyze them in vivo, which is only possible in mouse models. To ease the translation, we have genetically humanized models which will now allow a more accurate analysis of autoimmune mechanisms in human disease. In this review, we will also discuss what we know today regarding the targeted epitopes and the role of pathogenic and protective antibodies. With this knowledge as the basis, we have developed new types of serologic tests for RA.

## 1 The Horror Autotoxicity Dogma

Antibodies are central components of the adaptive immune system and are generally regarded as protective molecules that defend the host against infectious agents. Antibodies are soluble B cell antigen receptors produced by B cells differentiated into plasma cells. However, it is widely assumed that antibodies specific to self‐antigens are potentially pathogenic, leading to tissue injury and thereby contributing to the development of autoimmune disease.

The identification and role of antibodies were described in the late nineteenth century when Emil von Behring and Shibasaburo Kitasato demonstrated that serum from immunized animals could protect against diphtheria and tetanus. These studies introduced the concept of “humoral immunity,” in which soluble factors in blood mediated protection against infections. However, the observation that patients treated with horse serum antitoxins developed serum sickness indicated that antibodies could also be pathogenic, and the discovery by Karl Landsteiner of the ABO blood group system in 1901 demonstrated that naturally occurring antibodies could cause hemolytic transfusion reactions. The development of autoimmune theory represented a critical turning point. Early immunologists believed in the concept of “horror autotoxicus,” proposed by Paul Ehrlich, which suggested that the immune system normally avoided attacking self‐tissues, and evidence accumulated that self‐reactive antibodies could develop and contribute to disease. In the 1940s and 1950s, the occurrence of specific autoantibodies was found to be associated with autoimmune diseases such as systemic lupus erythematosus (SLE) and rheumatoid arthritis (RA). In the second half of the 20th century, the principles of immune‐cell selection were outlined, with T cells selected in the thymus and B cells in bone marrow. It was also found that T and B cells specific for self‐antigens were deleted, supporting the “horror autotoxicus” dogma. However, more recently it was also found that certain autoreactive T cells were selected in the thymus to be regulatory (Tregs), indicating that autoreactive lymphocytes could have a physiologic role [1].

A key issue was whether T and B cells needed to be stimulated through their antigen receptors to be selected in the central lymphoid organs. It was demonstrated that positive selection of alpha beta T cells imposes restriction to self‐major histocompatibility complex (MHC) molecules, ensuring that only thymocytes capable of recognizing the restricting MHC allele mature [2], which, along with the discovery of Tregs, suggested a positive selection of autoantigen specific T cells [3]. In the bone marrow, a corresponding selection through the B cell receptor, including the surrogate light chain, could be demonstrated [4, 5], whereas it was unclear whether a corresponding selection of antigen specific regulatory B cells is taking place.

**FIGURE 1:**
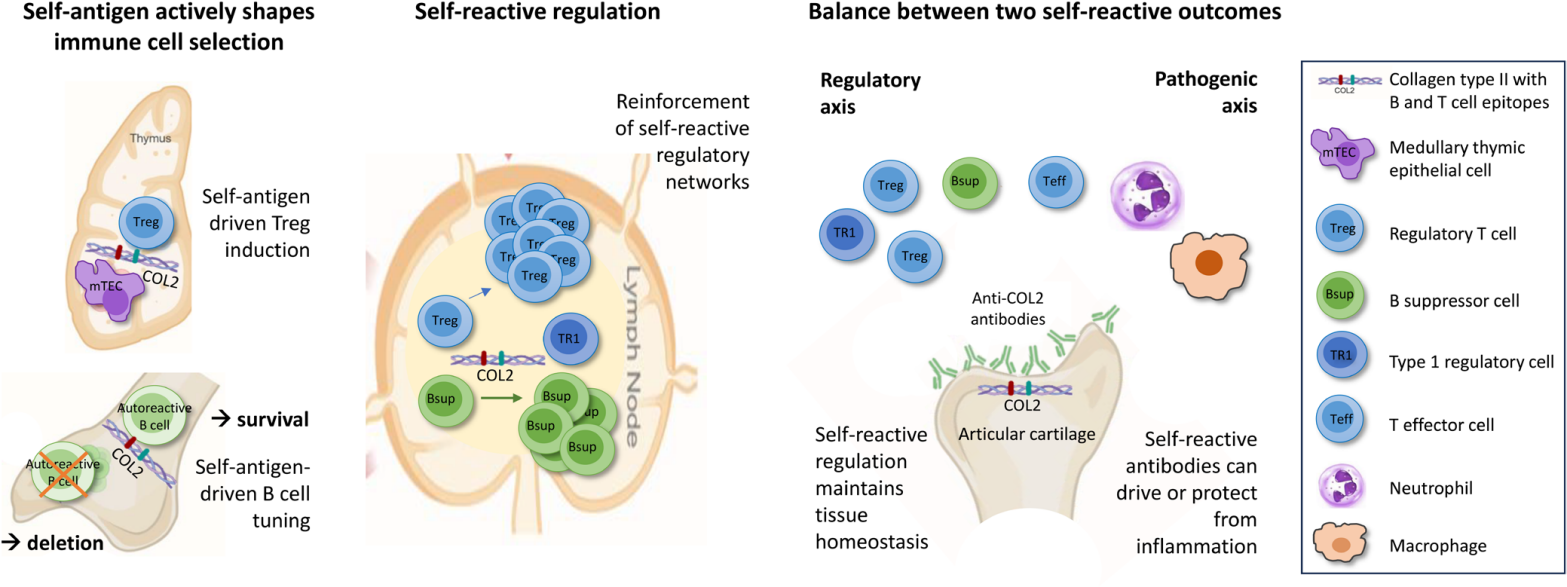
Self‐antigen‐driven selection of autoreactive lymphocytes and the balance between regulatory and pathogenic outcomes. This figure illustrates how self‐antigens shape the immune repertoire and establish regulatory networks essential for tissue homeostasis. Left: In central lymphoid organs, self‐antigens such as collagen type II (COL2) actively guide lymphocyte selection. In the thymus, COL2‐presenting medullary thymic epithelial cells (mTECs) induce the development of COL2‐specific regulatory T cells (Tregs). In the bone marrow, COL2‐binding B cells undergo central tolerance, where most autoreactive clones are deleted while a subset—including precursors of B suppressor cells (Bsup)—is positively selected, reflecting self‐antigen–driven B‐cell tuning. Center: In secondary lymphoid organs, self‐reactive regulatory networks are reinforced. Antigen presentation and cell–cell interactions promote the expansion of COL2‐specific Tregs and Bsup cells and support the differentiation of IL‐10‐producing type 1 regulatory T cells (TR1). These populations form a coordinated regulatory axis that shapes self‐reactive antibody responses and maintains peripheral tolerance. Right: In peripheral tissues, self‐reactivity produces two possible outcomes. The regulatory axis—comprising Tregs, TR1 cells, and Bsup—maintains tissue homeostasis by suppressing effector T‐cell activity and limiting inflammation. In contrast, the pathogenic axis arises when anti‐COL2 antibodies bind cartilage‐associated COL2 and recruit inflammatory effector cells such as neutrophils and macrophages. The balance between these regulatory and pathogenic pathways determines whether self‐reactivity supports tissue protection or contributes to autoimmune inflammation.

Since there are no foreign antigens during lymphocyte selection in the thymus or bone marrow, the only antigens that could stimulate the antigen receptors are self‐antigens, thus the immune repertoire is defined by autoreactivity. More recently, another view of the immune system is gradually getting more acceptance: that its origin lies not in the development of immune reactivity to infectious agents, but in the maintenance of tissue homeostasis through self‐reactive lymphocytes. Immune protection against infectious agents is then later superimposed on an immune system having a primary role in protecting tissues. With this view, the pathophysiologic role for B cells and antibodies can be analyzed through a new angle (Figure 1).

## 2 Selection of Autoreactive B Cells and Production of Autoantibodies

From an angle that the immune system develops to maintain tissue homeostasis, it is not surprising that a large part of the circulating antibodies is self‐reactive. These so‐called natural antibodies tend to be cross‐reactive but with a preference for interaction with certain antigens like DNA, immunoglobulins (Ig), polysaccharides, lipoproteins, but also collagen. It has been shown that the B1 cells are positively selected on self‐antigens [6] which could provide an explanation for the frequency of such antibodies. The roles of natural antibodies are, however, controversial but could encompass several different functions. They have been suggested to arise either from V(D)J (variable‐diversity‐joining) gene rearrangements that favor evolutionarily conserved pathogen‐associated structures or from selection on available antigens during B cell development. For example, collagen triple helical structures are rarely occurring in infectious organisms but are key components of tissues. An early observation was that an autoimmune response to the commonly occurring Type I collagen was difficult to generate, whereas an autoantibody response to the more cartilage tissue restricted Type II collagen (COL2) was strong and came early after immunization [7, 8]. Interestingly, COL2 is not only expressed in cartilage but also in central lymphoid organs. In the thymus, native unmodified COL2 is expressed in medullary thymic epithelial cells, resulting in negative selection but also in positive selection of COL2 specific Tregs, possibly dependent on posttranslational modifications [9]. Likewise, in the bone marrow, COL2 specific B cells escape negative selection and are maturing in the peripheral immune system to be regulatory by interacting and activating Tregs [10, 11]. These COL2‐specific B cells are likely important for protection against an autoimmune attack on joints and are present in not only mice and rats but also to the same frequency in healthy humans. If mice with COL2 specific B suppressor cells due to a V(D)J knock‐in are immunized with COL2 together with a strong adjuvant like complete Freund's adjuvant, they start to differentiate to plasma cells secreting arthritogenic antibodies [12, 13]. Based on these data, we propose that COL2 serves as a template antigen for selection of immune‐regulatory cells, together with a restricted set of tissue antigens that share similar properties—namely, expression in both the thymus and bone marrow in addition to expression in peripheral tissues.

Most autoimmune diseases are characterized by a general rise of antibody levels including natural autoantibodies, but a T cell dependent response could also generate an autoimmune response including more specific autoantibodies. Importantly, an increase of autoantibody levels often occurs years before the clinical disease onset, indicating that disease development starts with an autoimmune stimulation or reduced immune tolerance. One of the first autoantibodies found to be associated with an autoimmune disease was rheumatoid factor (RF) [14], that is, antibodies that bind modified IgG Fc, which was associated with rheumatoid arthritis (RA). RFs bind more strongly to allogeneic or xenogeneic IgG than to self IgG, and more strongly to enzyme‐cleaved IgG than to native IgG, consistent with preserved immune tolerance. However, the specificities of RFs are not well understood. Natural antibodies include clones with RF activity, but some RFs that accumulate somatic mutations become more specific. Rheumatoid factors develop years before arthritis onset and are associated with mucosal inflammation, for example triggered by smoking [15, 16]. Despite intense efforts over many years, the functional role of RFs remains unclear, and there is still no conclusive evidence for a pathogenic role. RF can also be detected in animal models of RA, although it remains unclear whether the fine‐specificity repertoire mirrors RA [17, 18]. Besides RA, it has long been known that RA sera bind to animal epithelial specimens—reactivities that were later defined to target the citrulline side chain; that is, antibodies to citrullinated proteins (ACPA) [19, 20]. Citrullination is a physiological posttranslational modification, but an antibody response to citrullinated proteins is strongly and uniquely associated with classical forms of RA.

## 3 Autoantibodies in Rheumatoid Arthritis

RFs are historically important for RA diagnosis, which is illustrated by being the only autoantibody included in the 1987 ACR Classification criteria for RA. Although widely used in RA diagnostics, they are not specific to the disease and may also be found in other rheumatic and infectious conditions, as well as in healthy individuals—particularly among older adults. Several pathogens can induce transient RF responses, suggesting a physiological role in host defense, in contrast to RF responses in autoimmune diseases which are typically more persistent [21]. RFs are included together with ACPA in 2010 classification criteria as well as the ACR/EULAR risk classification criteria for arthralgia at risk for RA [22, 23].

The estimated detection rate of RF‐IgM in early RA is 41%–66% of RA patients, while it is found in 62%–87% in established RA with meta‐analyses showing a pooled sensitivity of approximately 69% and a specificity of approximately 85%. These estimates vary depending on whether controls are healthy individuals, early undifferentiated arthritis, or disease controls with other rheumatic or infectious diseases [24]. Measurement of RF can be made with several methods, including nephelometry, latex agglutination, turbidometry, and ELISA‐based assays. Classic agglutination assays are simple and inexpensive but provide mainly qualitative or semi‐quantitative information, while ELISA or similar solid‐phase assays can also be adapted for detection of specific isotypes (IgM, IgG, and IgA). Despite technical advances, RF testing worldwide remains only partially harmonized, making it difficult to compare quantitative results from different assays [25]. While there is no global standard for the use of one detection method for RF, there are a series of international and national biological reference standards for RA serum, such as WHO W1066 or NIBSC 64/002 and 64/003. All three preparations originated from the same 1963 pool of serum from 197 RA patients which was split into 3 batches and used to calibrate laboratory assays to ensure consistent measurements of RF when diagnosing RA [26].

RF also has a prognostic value in RA; high titers are associated with a more severe disease phenotype. This includes higher disease activity, radiographic progression, extra articular manifestations, and a greater use of corticosteroids and biological disease modifying anti‐rheumatic drugs [27].

Taken together, the main strengths of RFs as biomarkers lie in the extensive clinical experience, broad availability, relatively good sensitivity, and prognostic association with a more severe disease phenotype. The main limitations are assay heterogeneity and a lower specificity compared to ACPA, in particular in comparison with clinically related diseases such as Sjögren's disease, systemic lupus erythematosus, or other inflammatory conditions. These limitations explain why RF has shifted from being the dominant serological marker of RA to being interpreted together with ACPA.

A class of antibodies showing more specificity for RA was reported in 1964 and named antiperinuclear factor (APF) [28]. This was followed by the identification of antikeratin antibodies (AKA) [29], which were later shown to bind to the protein filaggrin [30]. Studies using monoclonal antibodies against filaggrin demonstrated that APF and AKA were in fact the same RA‐specific antibody [31]. Despite their high specificity for RA, the inconvenient testing methods limited their usefulness as serological markers in clinical practice. It was not until the discovery that citrulline was the antigenic target that assays based on citrullinated peptides could be developed [20]. The first generation cyclic citrullinated peptide test (CCP1) was based on a single cyclic citrullinated peptide derived from filaggrin [32], which was followed by the second generation CCP test (CCP2) comprised of several synthetic citrullinated peptides which have not been publicly disclosed. Since its release in 2002, several attempts have been made to improve the detection of ACPA both by updated versions (CCP3, CCP3.1) and assays based on other citrullinated antigens such as vimentin and fibrinogen [33]. While some studies have shown modest improvements in the diagnostic accuracy from CCP3 or defined ACPA fine‐specific peptide assays, the vast majority of medical literature is based on CCP2 which is still used in many clinical labs. Since the introduction of the CCP2 test, ACPA has become a key serological marker incorporated into the 2010 ACR/EULAR classification criteria for RA as well as the ACR/EULAR risk classification criteria for arthralgia at risk for RA. For the diagnostic performance, CCP2 shows a very high specificity (90%–96%), with similar sensitivity to RF [34]. A limitation of the CCP2 test is the lack of negative control, but a new candidate reference preparation has been recently evaluated [35]. Furthermore, the abbreviation ‘CCP’ is used for different undisclosed peptides, making it difficult to evaluate and compare results across studies. Classical assays use citrullinated peptides to detect a polyclonal ACPA response in vitro, whereas the actual antigenic specificity in vivo is far more restricted because only a limited set of protein epitopes become citrullinated and accessible under physiological or inflammatory conditions. This discrepancy limits the biological interpretation of CCP‐based ACPA measurements.

Antibodies with reactivity to citrullinated proteins can also be detected in animal models, but the typical fine specificity and frequency like in RA are not seen in any animal models so far reported. Both RF and ACPAs have been detected many years before RA onset [15, 36, 37], arguing that the immune activation of these reactivities is connected to the etiology of the disease. However, like RF, the functional role of ACPAs is not known, although it has often been assumed that these antibodies play a pathogenic role. Since the sequences of the CCP2 assay are not public, it is not known if they overlap with any protein which may be citrullinated in vivo, making it difficult to study disease etiology and mechanisms of ACPA with the CCP2 assay. ACPA response as measured in the CCP2‐test should not be understood as a single antibody but as a family of reactivities directed against multiple citrullinated proteins, including fibrinogen, vimentin, alpha‐enolase, and Type II collagen [38].

Based on animal models, induced by immunization with cartilage derived proteins like Type II collagen (COL2), it has been found that such antibodies also occur in RA [39, 40, 41]. Although the occurrence of anti‐COL2 antibodies is found in only a few patients, they are, in similarity with antibodies causing collagen‐induced arthritis in animals, specific for triple helical epitopes on the COL2 molecule [42]. In addition, there is a high level of natural antibodies to collagen in both humans and mice [43, 44]. Importantly, however, the defined triple helical epitopes on COL2 contain arginine, which can be citrullinated [45]. A large part of ACPAs in RA interacts with such citrullinated COL2 epitopes, and many of these can bind to cartilage [46, 47]. Antibodies specifically reacting with triple helical COL2 appear to define a smaller subset of RA characterized by acute inflammatory onset around diagnosis [48] and are enriched in synovial fluid [41].

Among the autoantibodies not included in the 2010 classification criteria, anti‐carbamylated protein antibodies (anti‐CarP) are a stronger case as an adjunct marker [49]. In a recent meta‐analysis [50], anti‐CarP showed a pooled sensitivity of about 44% and specificity of about 96% for RA versus healthy controls; in ACPA‐negative RA, sensitivity remained low at about 24%, but specificity stayed high at about 95%. Anti‐CarP antibodies are present before arthritis develops [51]. It has, however, been difficult to determine the fine specificity of anti‐CarP antibodies. Most studies have used in‐house ELISA platforms and heterogeneous antigen preparations, which limits study comparability and slows clinical translation [50, 52, 53]. The best explanation for the detection of anti‐CarP antibodies is that ACPAs cross‐react to the homocitrulline sidechain, that is, a modification of the lysine sidechain which differs from citrulline by having 4 instead of 3 methylene groups. Antibodies uniquely reactive with carbamylated sites can be seen after immunization with adjuvants in mice but are different in specificities as compared with human ACPA [54].

## 4 Promiscuous Versus Private ACPAs

A major advance in the ACPA field came from moving beyond serum‐level assays to the isolation of monoclonal anti‐citrullinated protein antibodies. It was first done in mice; monoclonal antibodies against citrullinated fibrinogen or citrullinated collagen COL2 were raised by immunization and hybridoma approaches [55, 56]. In humans, monoclonal ACPAs have been recovered from citrullinated antigen‐specific peripheral blood B cells, single synovial fluid B cells, synovial plasma cells, and circulating plasmablasts [57, 58, 59]. Together these studies established that ACPA positivity does not represent a single antibody specificity, but rather a family of clonally distinct antibodies with major differences in fine specificity, cross‐reactivity, and biological activity measured in vitro. This perspective is essential to understand in more detail because standard anti‐CCP assays collapse a highly heterogeneous repertoire into one composite serological readout.

One useful way to classify ACPAs is according to the breadth of antigen recognition. At one end are private antibodies that uniquely bind a citrullinated epitope, thus dependent not only on the citrulline side chain but also the surrounding amino acids. At the other end are promiscuous antibodies that specifically bind only the citrulline side chain, which could be present on many diverse epitopes [46, 60, 61, 62]. Importantly, this promiscuity is not simply low‐affinity stickiness. Rather, many clones recurrently recognize citrulline‐centered amino acid motifs presented in compatible structural contexts [63]. The antigenic unit is therefore often a motif on a cyclic peptide rather than an individual source protein. This concept helps explain why the serum ACPA response can appear to spread from one antigen to another while still rooting in a restricted set of paratope solutions.

Structural and lineage analyses provide a mechanistic basis for this behavior. Crystallographic work on the ACPA clone E4 showed that the citrulline sidechain binds in a unique pocket, with only limited interactions with surrounding side chains [61]. Such a binding mode readily accommodates different proteins carrying analogous citrullinated motifs and is often misinterpreted as crossreactivity, but rather mimics anti‐hapten antibodies with promiscuous reactivity [63]. Consistent with this, affinity maturation does not simply strengthen binding to a single citrullinated target, but modifies its selectivity to bind to citrulline on different peptide backbones [59]. ACPAs also display unusual molecular features, including variable‐domain N‐linked glycosylation sites and reduced surface charge, suggesting that noncanonical selection pressures could influence the autoreactive B cell compartment [64]. All so far crystallized human ACPAs show the same promiscuous type of binding pattern as well as Fab glycosylation [62].

Recognition of peptides in solid phase assays is only one layer of ACPA biology. A second and functionally more relevant axis is whether a given clone can engage disease‐relevant tissue antigens and trigger effector pathways. In mice, monoclonal antibodies specific to citrullinated COL2 can bind inflamed synovium or arthritic cartilage and enhance or induce arthritis [55, 56]. With autoantibodies, including ACPAs, derived from humans, there are many reports describing different pathogenic effects in cellular systems in vitro but we are not validating these results here as the important issue is their effect in vivo. Large amounts of sera isolated from RA patients have been reported to induce arthritis in mice [65], but it has been difficult to induce disease in mice with well‐defined autoantibody clones derived from humans. In contrast, induction and enhancement of arthritis with monoclonal antibodies binding specifically in vivo to cartilage antigens, or to citrullinated COL2, are straightforward [7, 56, 66, 67, 68, 69, 70]. Similar effects with human promiscuous ACPAs or RFs have not yet been shown. Transfer of ACPAs has been shown to induce bone erosion, but this effect is not dependent on their citrulline specificity but rather their ability to engage Fc‐receptors through immune complex formation [71].

These observations argue that pathogenicity is not an intrinsic property of ACPAs but emerges when antibodies can specifically target cartilaginous joints in vivo, involving an inflammatory environment that permits inflammation inducing immune‐complex and Fc receptor signaling. Antibodies corresponding to the mouse‐derived pathogenic anti‐cartilage antibodies, including ACPAs binding citrullinated COL2, are common in RA but have not yet been clonally isolated. Thus, we have reasons to believe that among the autoantibodies typical of RA, there are pathogenic clones which presumably appear before clinical onset and are sustained throughout the disease course.

## 5 Protective ACPAs; Underlying Mechanisms

For decades, the central “horror autotoxicus” dogma of the role of autoantibodies has positioned RF and ACPA as pathogenic autoantibodies. However, after testing ACPA clones for functional importance in vivo, using mouse models, it has become clear that many of the well‐defined promiscuous ACPA clones derived from RA patients protect against development of arthritis, even clones previously reported to be pathogenic in vitro. For the first observation, we used the E4 antibody that was defined as a highly promiscuous ACPA [72, 73]. Antibodies with similar specificity are commonly occurring in RA. After i.v. injection, the E4 antibody could protect mice from several different types of autoimmune arthritis models, including collagen antibody induced arthritis and the T cell dependent glucose‐6‐phosphatase peptide induced arthritis. It operates by interacting with COL2 on the cartilage surface and citrullinated epitopes on alpha enolase (ENO1), forming immune complexes that activate the inhibitory FCGR2B on synovial macrophages. Studies using previously reported monoclonal ACPAs confirmed that many of these antibodies are protective [74, 75]. However, the fine specificity of the protective antibodies is diverse, and it is likely that protection could be mediated by different downstream mechanisms. Of particular importance are antibodies to citrullinated histones, which had earlier been shown to block NETs formation by neutrophilic granulocytes [76], and it could be shown that this specificity is commonly occurring in RA [75]. A third class of antibodies with arthritis protection potential are antibodies to certain epitopes on triple helical collagen. Antibodies to triple helical collagen are in general epitope‐specific and induce arthritis after binding to cartilage, but with one exception. Antibodies to the collagen crosslinking site, the F4 epitope, do not induce arthritis and, when injected together with arthritogenic antibodies, they protect against arthritis [77]. These antibodies are also occurring in RA but are less frequent than ACPAs and clearly represent an additional arthritis protective mechanism.

**FIGURE 2:**
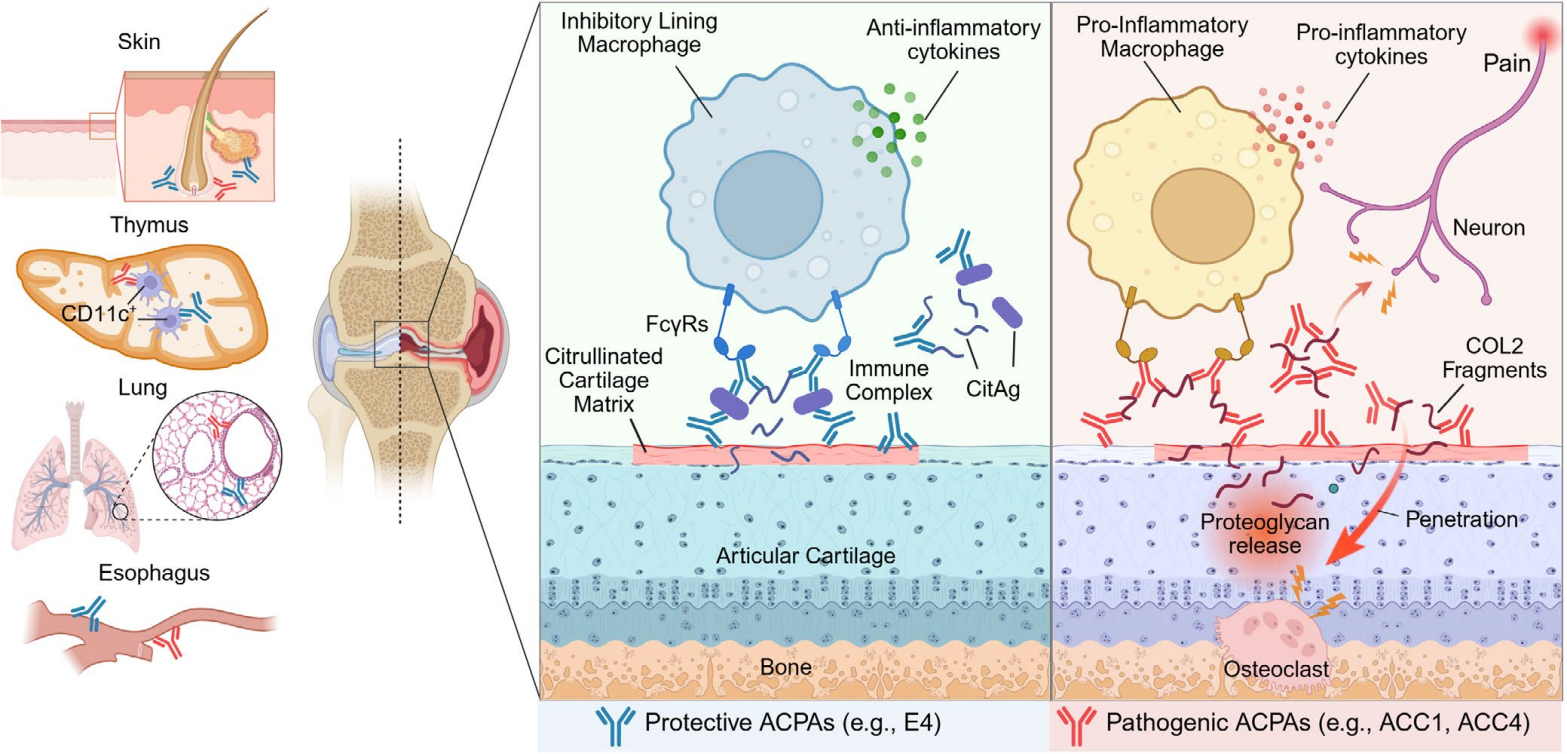
Functional dichotomy of protective versus pathogenic ACPAs in non‐joint tissues and the joint microenvironment. Left panels illustrate the in vitro binding of protective (blue) and pathogenic (red) ACPAs across mucosal and barrier sites, including skin, thymus (CD11c + cells), lung, and esophagus, demonstrating that both antibody subsets can bind widely to these tissues. However, the in vivo binding of these antibodies, especially for protective ACPAs, are likely restricted, or have minimal functional effect on other tissues, as physiologically citrullinated antigens (e.g., citrullinated filaggrin) are commonly expressed in various tissues, but the protection is specifically against arthritis in vivo. For the pathogenic ACPAs, the main target remains to be citrullinated or native COL2 in the joint, with a similar broadened staining on other tissues in vitro, which may be due to the exposure of certain citrullinated epitopes resembling the citrullinated COL2 epitopes. On the right panel, the magnified inserts contrast their intra‐articular mechanisms: Protective ACPAs (e.g., clone E4) form immune complexes with citrullinated antigens (CitAg) originating from RA‐related proteins such as alpha‐enolase (ENO1) and COL2 to engage macrophage Fc receptors (e.g., FCGR2B), driving anti‐inflammatory cytokine release and preserving tissue homeostasis. Conversely, pathogenic ACPAs (e.g., clones ACC1, ACC4) trigger pro‐inflammatory macrophage activation and engage the peripheral nerve endings of dorsal root ganglion (DRG) neurons to mediate pain signaling (right). The pathogenic cascade also induces matrix degradation, the release of proteoglycans and subsequent fragmentation of COL2 fragmentation, potentially breaching the articular cartilage barrier to allow deep immune complex penetration into the subchondral bone, where they accelerate osteoclast‐mediated bone resorption or osteoclastogenesis. ACPA, anti‐citrullinated protein antibodies; COL2, collagen type II; FCGR2B, Fc gamma receptor IIb.

The underlying mechanisms driving these divergent protective mechanisms are not fully understood. They are all dependent on their antigen specificity, that is, the combining site in the Fab fragment as well as the FcR binding determined by their Fc part. Thus, the downstream mechanisms are likely regulated through how and where immune complexes are formed. Figure 2 illustrates possible mechanisms of protective and pathogenic antibodies.

The only protective type of antibodies with a classical promiscuous ACPA activity is represented by the E4 antibody. The E4 antibody binds to a large number of different citrullinated peptides in vitro but has a very limited set of targets in vivo; it binds to macrophages and dendritic cells but also to joint cartilage [73]. In vitro histological staining showed that E4 could also bind strongly to tissues including lung, esophagus, skin, and thymus, displaying classical ACPA reactivity. However, whether such binding could also be observed in vivo remains to be determined, but it is unlikely to have a significant effect on the tissues, since the arthritis protection is joint‐specific and the antibody did not impact, for example, the experimental autoimmune encephalomyelitis or psoriasis models. Proteomic analysis of macrophages and synovial fluid shows a restricted set of proteins interacting with E4 including citrullinated ENO1. The E4 antibody induces FCGR2B expressing macrophages to secrete IL10 provided they are co‐incubated with citrullinated ENO1, indicating activation of regulatory functions. Taken together, these observations led us to hypothesize that E4 mediates its protection by activation of regulatory lining layer macrophages in contact with the cartilage surface. The Fab‐glycan did not influence the protective effect, but Fc‐glycan is crucial, probably due to its importance for binding FCGR2B. An important remaining question is why the E4 immune complex preferentially interacts with FCGR2B and not with the activating FCGR3. Possibly, it could be that the inhibitory macrophages preferentially express FCGR2B. It is important to note, however, that the E4 antibody also induced a long‐lasting protection of glucose‐6‐phosphatase peptide induced arthritis, which is a T cell dependent arthritis with no known effects of pathogenic antibodies. Thus, additional protective mechanisms may operate, like changing the function of dendritic cells in the thymus that is targeted by E4.

Clearly, several different protective pathways operate, demonstrated by the collagen reactive F4 type of antibodies. One way for the F4 antibodies to protect against arthritis is to delete neutrophils through the binding to FCGR3 (the human homologue is FCGR2A), an effect that operates well even in mice deficient in FCGR2B. The specificity of the F4 antibodies is to bind to the triple helical collagen epitope around the crosslinking site. However, these protective antibodies also cross‐react with other selected triple helical collagen epitopes, including C1q. Triple helical collagen is also used by several C‐type lectin receptors, although it is not yet clear how these interactions lead to arthritis protection. Although the typical F4 antibodies are only infrequently occurring in RA sera, similar types of reactivities are common in the repertoire of natural antibodies which may indicate that such regulatory antibodies could have an important physiological role. Yet another mechanism for arthritis protection has been provided by antibodies made from phage display [76], which target citrullinated histones. These antibodies block neutrophil extracellular trap (NET) formation, leading to the phagocytosis of the entire complex by macrophages and suppressing NETosis induced joint inflammation.

The functional evidence supporting ACPA‐mediated protection is currently derived from a highly restricted pool of monoclonal antibodies and relies on murine models of induced arthritis. The gap between the speculations from the highly controlled, genetically homogenous mouse models and the profoundly heterogeneous landscape of human seropositive RA remains to be filled. It is for example possible that the function in vivo of protective antibodies could be modified by other autoantibodies like rheumatoid factors, which could change the key function of inhibitory immune complexes.

## 6 Pathogenicity of Monoclonal ACPA; Underlying Mechanisms

Mouse models induced by defined antibodies to cartilage antigens like COL2 (CAIA) have been used to understand how arthritis is induced. After injection, the antibodies rapidly bind to cartilage, but in a normal mouse, development of clinical arthritis takes at least 48 h. The delay reflects the time needed for complement activation, Fc gamma receptor signaling, neutrophil recruitment, and the cytokine burst as well as the protective effect of synovial lining layer macrophages expressing the inhibitory receptor FCGR2B. Inflammatory proteases activated downstream subsequently deplete proteoglycans, destabilize cartilage, and release potentially inflammatory COL2 fragments, thereby opening cartilaginous joints for an inflammatory attack and amplifying the progression of arthritis [78, 79, 80, 81, 82, 83].

To be arthritogenic, antibodies need to specifically bind cartilage in vivo. The only ACPAs shown to induce arthritis are derived from mice and have a high specificity to cartilage in vivo [56]. In contrast, none of the promiscuous ACPAs have been identified to have the same properties, some of them may even exhibit protective effects by binding to the cartilage. Clinical arthritis is, however, not the only pathogenic trait in RA. A persistent clinical paradox in RA is that patients frequently report joint pain even when the presence of inflammatory markers is minimal. Arthralgia, that is, joint pain, is not strictly associated with arthritis and often appears before the clinical arthritis onset and may also sustain after the decline of active arthritis. In addition, a typical sign of RA is bone erosion that could also develop independently from clinical arthritis. Thus, these traits may be dependent on different pathogenic mechanisms. Cartilage specific antibodies induce not only arthritis, but also joint pain [84] and bone erosions [70]. Cartilage specific antibodies, including ACPAs with specific cartilage binding, induce pain even before arthritis develops and with a sustained effect after the acute phase of arthritis. In contrast, the effects of promiscuous ACPAs are controversial, but well characterized antibodies like E4 do not induce pain or erosions [73, 84]. In a recent study on pain induced by cartilage specific antibodies, including both COL2 specific and private ACPA (to citrullinated COL2), an induction of Type I interferon (IFN‐I) was attributed to consistently activating the MNK1/MNK2–eIF4E pathway that sensitized the joint‐innervating sensory dorsal root ganglion (DRG) neurons, subsequently leading to pain [85].

Even though the so far tested promiscuous ACPAs are either protective or have no detectable pathogenic effect, there are other subsets of ACPA that are pathogenic. As earlier described, based on the interaction with citrullinated epitopes of several crystallized monoclonal ACPAs [56, 61, 86], ACPAs could be classified as promiscuous (e.g., clone E4), private cross‐reactive (e.g., clone ACC1) and private specific (e.g., clone ACC4) [63]. Obviously, there is no sharp distinction between promiscuous (i.e., with negative influence by surrounding amino acids) and private (i.e., with positive interactions with surrounding amino acids), but a gray scale. Unlike the protective ACPA E4 which was derived from an RA patient, the murine clones ACC1 and ACC4, which were derived from mice immunized by COL2 or peptidyl arginine deiminase 4 (PAD4)‐treated citrullinated peptides containing B and T‐cell epitopes, were shown to be pathogenic by directly inducing arthritis, or by enhancing the disease severity in combination with cartilage antibodies [56, 86]. Although both private ACPAs bind citrullinated COL2, they target different modifications of the same epitope. ACC1 binds to citrullinated COL2 and ACC4 with citrullinated COL2 alpha chains. However, both bind esophagus as in the classical assay for ACPA. ACC1 could cross‐react with the native epitopes, enabling its direct binding to naïve joint cartilage. This localized binding forms immune complexes on the cartilage surface, which subsequently activates the complement pathway and recruits inflammatory macrophages, initiating joint inflammation and damage. On the contrary, ACC4 binding requires that cartilage is already destabilized and COL2 citrullinated, explaining why the antibody does not induce arthritis but only enhances arthritis initiated by other antibodies. Therefore, cartilage binding ACPAs such as ACC1 and ACC4 behave in ways like other autoantibodies targeting specific joint proteins to induce joint inflammation, largely depending on the interaction between ICs and Fc receptors as well as the complement system [78]. However, although it is likely that human derived antibodies could be pathogenic, there is no direct proof. In RA sera there are both promiscuous and more private ACPA, including a large proportion binding to joint cartilage.

A major problem is that we do not have animal models that develop a corresponding disease like RA, including the unique production of ACPA. It is possible that testing of antibodies in a mouse which already has a full set of the RA autoantibodies, with both ACPA and RF, could give different results as the downstream, both protective and pathogenic effects, are dependent on the fine‐tuned action of immune complexes.

## 7 Detection of Pathogenic and Protective ACPA in Serum of RA

ACPAs appearing in circulation several years before RA onset have a widespread reactivity to citrullinated peptides. Several ACPA fine specificities, including ACPAs against citrullinated epitopes of alpha enolase (ENO1), PAD4, citrullinated vimentin 2, histone 1, fibrinogen, and filaggrin, appear already in presymptomatic individuals preceding RA symptoms or diagnosis [38, 87, 88]. ACPAs that precede RA are heterogenous and recognize a broad range of endogenous and exogenous citrullinated antigens [89]. Epitope spreading closer to RA onset is well‐established [38, 90, 91, 92], and rising ACPA levels—used in both RA classification and prognosis [93]—likely reflect this progressive diversification of antibody response. In a recent meta‐analysis, arthralgia in combination with CCP2 and IgM‐RF led to the fastest rate of progression to RA [94] and the extent of the repertoire is associated with a higher risk of developing arthritis in patients with seropositive arthralgia [91].

Clinically suspected arthralgia (CSA) is a symptomatic at‐risk stage which may develop into RA or resolve spontaneously. The cellular and humoral mechanisms of spontaneous resolution of CSA are poorly understood while causal relation of the subclinical inflammation and the phenotypic presentation of CSA has been suggested [95]. The ability to predict resolution of CSA with biomarkers would be clinically relevant in the management of patients with CSA both economically and in terms of healthcare resource utilization. A recent study reported that more than half of both ACPA‐positive and ACPA‐negative patients with CSA (59% and 71%, respectively) achieved spontaneous resolution of CSA within 2 years follow‐up [96], pointing toward minor roles of ACPA, measured by anti‐CCP2, in predicting CSA resolution. Whether sensitive assays to determine the pathogenic and protective ACPA fine specificities would improve the accuracy of prediction remains to be shown.

Specificity in serological assays is essential for distinguishing RA from other joint inflammatory diseases. We have used peptides derived from disease‐associated proteins in joints—in either native or post‐translationally modified forms—to capture RA‐related antibody responses in serum. These assays detect autoantibodies that discriminate early RA from population controls, as well as from psoriatic arthritis (PsA) and osteoarthritis (OA), with high accuracy. Importantly, they also identify seronegative early RA (anti‐CCP2 and RF negative) with high specificity in several independent early RA cohorts [97, 98]. Autoantibodies to disease‐associated proteins in joints and ACPA fine specificities binding to citrullinated epitopes on peptidyl arginine deiminase 4 (PAD4) and alpha‐enolase (ENO1) were shown to identify a large proportion of pre‐symptomatic individuals later developing RA from population controls [87, 98]. A proportion of presymptomatic seronegative individuals (anti‐CCP2 and RF negative) who later develop RA were identified by the SeNe assay [98], indicating potential clinical utility for early diagnosis of RA and, importantly, for selecting candidates for preventive intervention.

Specific autoantibody signatures have been linked to better outcomes in RA. In early RA, ACPAs were found to associate with low numbers of affected joints [99]. We have shown that certain ACPA fine‐specificities associate with lower disease activity in treatment‐naïve early RA [87], and that autoantibodies against joint‐related proteins, in combination with male sex, could serve as predictive markers for remission with high specificity [100].

Specific autoantibody signatures have been linked to worse outcomes in RA. Autoantibodies directed against joint‐related proteins associate with structural damage. Antibodies to GPI(293–307) peptide were detected more frequently in early RA than in population controls and predicted RA onset within a year in presymptomatic individuals [69]. Higher levels of these anti‐GPI (293–307) antibodies were also associated with radiographic joint damage in early RA. Among anti‐modified protein antibodies (AMPAs), anti‐CarP antibodies have been associated with more severe radiographic progression in both the total and in ACPA‐negative RA [101]. Combined analysis of AMPA responses to carbamylated, acetylated, and citrullinated epitopes improved prediction of future radiographic progression compared with ACPA alone; notably, patients positive only for ACPAs did not progress more than those negative for all AMPAs [102]. Regarding ACPAs fine‐specificities, antibodies to citrullinated COL2 and glucose‐6‐phosphate dehydrogenase peptides increased diagnostic specificity in new‐onset RA, whereas CCP positivity alone predicted joint destruction with low specificity [100].

Increased cardiovascular mortality in patients with RA is well established [103]. There is evidence that ACPAs are associated with increased overall mortality in patients with RA, including cardiovascular mortality; however, these associations may be influenced by confounding factors such as disease activity and contemporary pharmacotherapy [104, 105]. A recent study showed that RA‐associated inflammation (higher C‐reactive protein (CRP) levels) was a determining confounding factor behind the link between ACPA and cardiovascular mortality in patients with RA [106]. When it comes to comorbidities and mortality in RA, the role of protective and pathogenic ACPAs in the risk of developing comorbidities and the shorter life expectancy in patients with RA remain to be dissected.

Taking together, detection of pathogenic and protective ACPA in RA may provide more information than an ACPA test alone for several purposes including resolution of CSA, prediction of RA onset, prognosis, joint destruction or treatment response. Validation of the described ACPA fine‐specificities and discovery of novel ACPA fine specificities and their detailed characterization are needed to confirm their clinical utility as well as their contribution to priming, disease onset and regulation of autoimmunity as well as disease progression.

## 8 Humanization of Mouse Models for Studies on Autoimmunity and ACPAs

Classical animal models such as CIA, induced by COL2 immunization, develop high titers of pathogenic anti‐COL2 antibodies, but with very low titers of COL2 specific ACPAs and no RA typical promiscuous ACPAs. In contrast, RA has high titers of ACPAs, including ACPAs cross‐reacting to cartilage; only a few patients have antibodies specifically binding cartilage‐like antibodies to native triple helical COL2. The explanation for this discrepancy is likely to be multifold. In RA, the inducing factors are unknown whereas in mice, arthritis is induced by immunization with a cartilage protein, like COL2. Genetics are also different, although the strong MHCII association with different DRB1 alleles with some shared peptide binding structure [107]; in RA it is suggested to be certain alleles of DRB1*04 (*04:01, *04:04, *04:05) whereas in the mouse it has been shown that the MHCII allele A^q^ causes CIA [108]. Interestingly, mice expressing DR*04:01 develop CIA after immunization with COL2 since they bind the same COL2 peptide as A^q^ [109, 110, 111].

Humanized mouse models should become an essential tool for studying autoimmune diseases because they bridge a fundamental gap between human and conventional animal research. On one hand, clinical studies provide invaluable insights into disease heterogeneity, immune signatures, and clinical progression, but they are inherently observational. Ethical and practical constraints limit mechanistic interrogation, temporal resolution, and experimental manipulation of specific pathways in humans. As a result, causality often remains difficult to establish except for genomic studies. An additional problem is that even in human genetic association studies of complex diseases it is not possible to position the gene due to linkage disequilibrium [112]. In mice it is possible to clone genes and perform experiments, as discussed in a recent review [113], but traditional mouse models do not fully recapitulate the human immune system. Importantly, different experimental settings, as compared with the clinical situation (acute vs. chronic, inducing environmental factors), but also flaws in experimental design, are likely major causes explaining differences [114]. Obviously, there are also species differences, and although functional pathways are largely conserved, the used genes and proteins may differ as well as molecular targets. These differences concern in particular the specificity of the adaptive immune system profiled by the major histocompatibility complex Class II (MHCII) molecules.

The challenge is to humanize the mouse without disturbing its normal physiology. Obviously, we need to select the key human genetic factors and insert them to fit into the mouse context. This has obviously failed with the humanized stem cell transfer models which create a non‐physiologic dysregulation in the mouse. It has also failed due to the inherent unpredictability of transgene integration and expression. Pronuclear injection typically results in random genomic insertion, which can disrupt endogenous genes or regulatory regions and lead to position‐effect variegation, causing inconsistent or non‐physiological expression patterns. Transgenic expression of human MHCII genes, for example, led to aberrant expression resulting in toxicity of B cells [115] and incomplete expression led to lack of thymus selection [116]; it is also likely that, in addition, many artificial epistatic interactions due to discrepancies between mouse and human proteins occurred. These abnormalities cause human MHCII transgenic mice to have weak immune tolerance, leading them to mount artificially strong autoreactive responses. In terms of potential ACPA responses, there is no evidence so far that DR*04:01 transgenic mice develop ACPAs with the same specificity as in human RA although it is possible to induce antibody reactivity to citrullinated peptide, but they are not different from WT controls [117].

Proper humanization of mice benefits from clearly defining the human gene variants relevant to disease development and immune selection, and from careful consideration of any potential epistatic interactions. We have established humanized mouse strains with proper expression and function of human genes with polymorphism likely to be of major importance for the development of autoimmune disease [116, 118]. Firstly, we used a standardized mouse strain, the C57Bl6/N mouse, and introgressed the *Mus musculus musculus* derived Cia9i quantitative trait loci (QTL) containing the FcgR3/FcgR2b locus, with polymorphism causative for the development of arthritis [119]. The original QTL in the C57Bl6/N strain derived from the *Mus musculus molossinus* strain instead and is protective against arthritis. This introgression replaced the protective C57Bl6/N allele with the arthritis‐susceptible *M. m. musculus* variant, yielding a strain carrying the appropriate susceptibility‐associated FCGR region QTL. A corresponding QTL has also been discovered to be associated with autoimmune diseases in humans [120]. Secondly, RA‐relevant MHCII genes were knocked into the modified B6 strain. This led to expression of chimeric mouse—human molecules with the ectodomains of human MHCII alleles; DRA and DRB1*04:01 (common in European population and associated with RA), DRB1*04:02 (not associated with RA) or DRB1*04:05 (associated with RA in the east Asian population). These were expressed together with chimeric mouse—human CD74 (invariant chain), CD4 and CD223 (LAG3) molecules. Together these molecules mimic a human immune response [118].

Interestingly, the DRB1*04:01 expressing mice (denoted Parker if combined with CD74 and Primus if combined with CD74, CD4 and CD223) are highly susceptible to collagen induced arthritis with low but significant titers of ACPA, in contrast to mice expressing DRB1*04:02 [116]. Accordingly, HLA‐DRB1*04:01 mice also developed higher titers of autoantibodies to COL2 compared to HLA‐DRB1*04:02 controls. These findings indicate a strong and dominant role of HLA‐DRB1 alleles in both autoantibody generation and the progression of arthritis.

**FIGURE 3:**
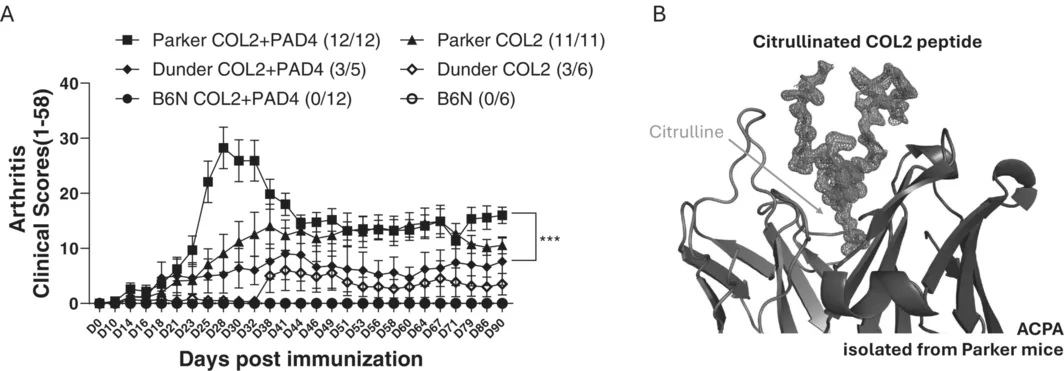
Humanized mouse model expressing human RA susceptibility allele HLA‐DRB1*04:01 develop severe arthritis and develop an ACPA response. (A) Parker mice expressing the human RA susceptibility allele DRB1*04:01, DRA and human invariant chain CD74, develop more severe arthritis than Dunder mice (expressing allele DRB1*04:02, DRA and human invariant chain CD74) when immunized with COL2 complexed with a minimal amount of PAD4 or mice without the human allele (B6N) or when immunized with COL2 alone. Statistics were determined using 2‐way ANOVA with Sidak's multiple comparisons test. The number of arthritic mice/total number of mice are indicated in parenthesis. (B) A monoclonal ACPA (clone LN20‐D9), derived from the Parker mouse, binds a COL2 citrullinated peptide and essentially interacts only with the citrulline sidechain as a typical promiscuous ACPA, as illustrated by the crystal structure. ACPA, anti‐citrullinated protein antibody; COL2, collagen type II; HLA, human leukocyte antigen; PAD4, peptidyl arginine deiminase 4.

Adding small amounts of PAD4 to the immunization enhanced arthritis and ACPA development [118] (Figure 3A). ACPA clones were isolated and shown to specifically bind the citrulline side chain in a promiscuous way [118] (Figure 3B). This suggests a more physiologically relevant arthritis model that better resembles human RA, in which ACPA is a key serological hallmark. Such models would be optimal to dissect the function of autoantibodies in vivo, particularly ACPAs or other autoantibodies derived from humans. They allow direct testing of the pathogenic versus protective antibodies in a setting that closely mirrors human RA, including allele‐specific immune responses and PAD4‐dependent citrullination. For instance, recombinant antibody R69‐4 demonstrated protection in Primus mice following COL2 immunization, but with a different magnitude than in A^q^ mice [121]. This suggests a difference between mouse autoimmune response and humanized autoimmune response that could be utilized to establish causality, track ACPA dynamics over time, and evaluate targeted therapeutic strategies with high translational relevance.

Another example of disease‐relevant targeted humanization is the introduction of the single‐nucleotide polymorphisms (SNP) in human neutrophil cytosolic factor (NCF1) that replaces arginine with histidine at position 90, a variant that is associated with several autoimmune diseases including SLE and RA [113]. In this strain, all mice developed severe lupus if infected with murine norovirus, a single RNA virus with pathology very similar to that of human norovirus [122]. These mice also developed autoantibodies typical of SLE after norovirus infection. Thus, these humanized mice mimic complex human autoimmune diseases and enable detailed mechanistic analysis.

## 9 Future Diagnostic Directions Based on Autoantibodies or ACPA

**FIGURE 4:**
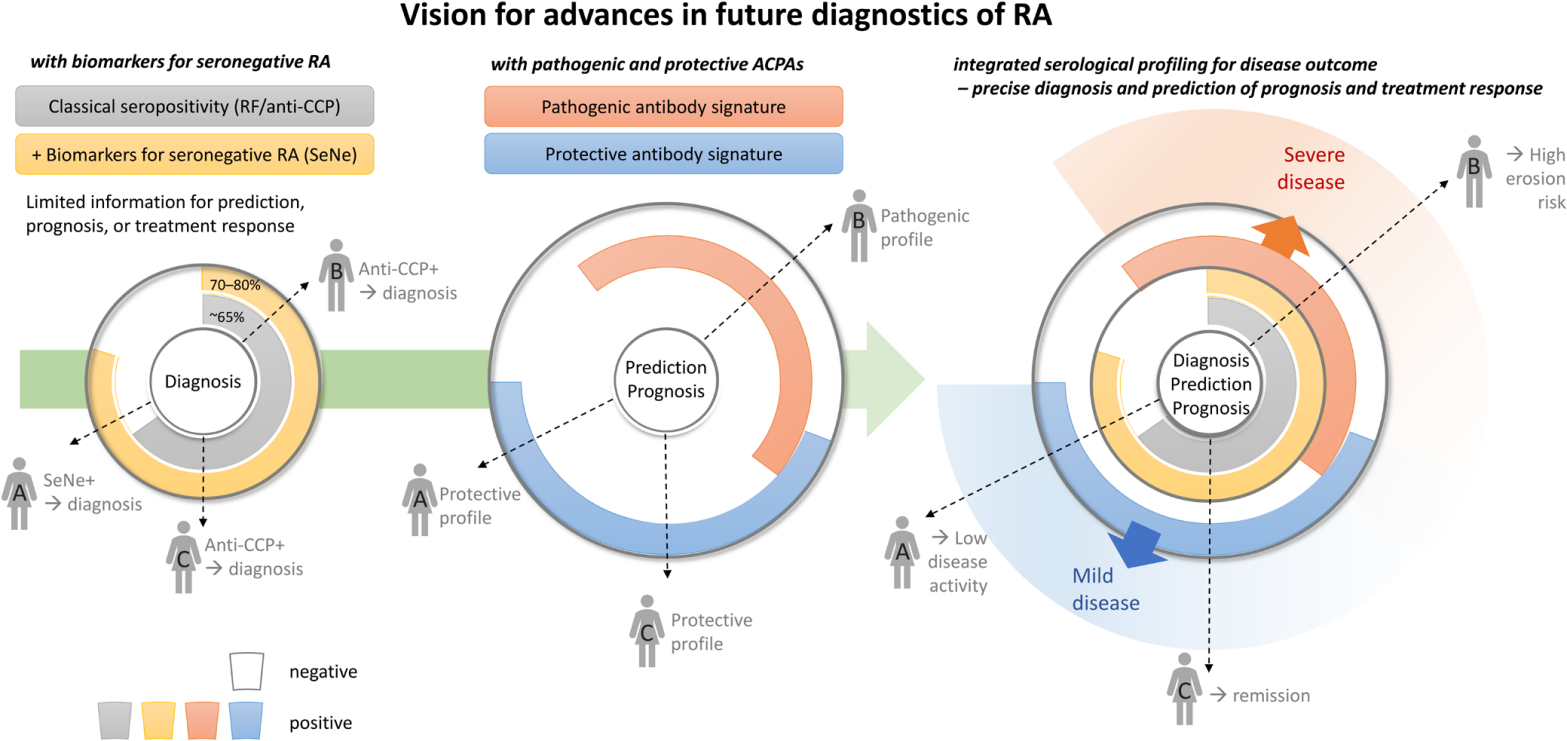
Conceptual diagnostic model integrating protective and pathogenic ACPA signatures with classical serology and emerging biomarkers for seronegative RA. Each concentric ring represents a diagnostic marker layer, and the colored arc segments indicate the approximate fraction of early RA patients positive for that marker type. Each radial line corresponds to an individual patient; a patient is positive for a given marker category when their radius intersects with the colored segment of that ring. Protective and pathogenic ACPA arcs occupy different angular sectors, reflecting their limited co‐occurrence. Three examples of patient radii (Patients A, B, and C) illustrate how different biomarker combinations map onto the diagnostic layers. Protective and pathogenic ACPA signatures shift predicted disease trajectory toward milder or more severe outcomes, respectively. SeNe, novel biomarkers for SeroNegative RA [97, 98] are examples of candidate diagnostic tests that identify a proportion of classically seronegative (anti‐CCP‐negative and RF‐negative) early RA patients. ACPA, anti‐citrullinated protein antibodies; CCP, cyclic citrullinated peptide; RA, rheumatoid arthritis; RF, rheumatoid factor.

Novel biomarkers in rheumatology aim at improving diagnosis and prediction of RA to allow all patients with early RA to catch the window of opportunity for effective treatment [123]. The different aims for new diagnostics of RA are illustrated in Figure 4. Markers that narrow the serological gap by identifying classically seronegative (anti‐CCP and RF negative) early RA patients would help confirm the diagnosis without delay. High specificity of such tests would reduce misdiagnoses between RA and other joint inflammatory diseases such as osteoarthritis. Prognosis of erosion severity in newly diagnosed RA could be improved by profiling the balance of the protective and pathogenic antibodies, and such signatures might even prove useful in predicting the likelihood of remission or treatment unresponsiveness. Integrating sensitive and highly RA‐specific biomarkers with antibody profiling could become standard for describing ongoing cellular and humoral processes in those with new‐onset RA as well as individuals with suspicion of or risk for the disease.

The focus in identification of novel biomarkers has been on classically seronegative (RF and anti‐CCP negative) for a reason; this remains an unmet need in RA [124]. Prediction of RA onset and prognosis of early RA aims to allow early intervention. Most often, ACPA‐positive individuals are recruited to research studies to predict RA onset due to the relatively high likelihood for development of the disease soon. Importantly, clinical prognostic factors are often the basis of prognosis, but there is room for improvement where autoantibody profiling could fit in. Autoantibodies associated with radiographic progression in early RA would be of help in guiding treatment decisions. Furthermore, the influence of confounding factors such as RF or body mass index (BMI) should be considered when such associations are validated or risk algorithms of joint destruction are established. Prediction of resolution of CSA by determining the prevalence of pathogenic and/or protective ACPA is another possibility that could aid in selecting patients for trials investigating the efficacy of a therapy in preventing arthritis in the pre‐clinical phase of rheumatoid arthritis. Besides commercial risks, costs, and timelines of required clinical performance studies may limit the development of predictive in vitro diagnostic (IVD) devices. While samples from patients with early RA are often available, samples from asymptomatic individuals are rare and the proportion of at‐risk for RA patients developing RA within a short timeline is small. This will create commercial uncertainties for developers, especially if the predictive or prognostic value is limited, and may thus delay the market entry.

Detection of protective and pathogenic ACPA in circulation to generate individualized fingerprints for dominance of either protective or pathogenic signatures may be feasible using multiplex IVD assays. Feasibility wise, it requires minimal extra efforts to use the same samples collected for the detection of current serological RF and anti‐CCP antibodies. Furthermore, no extra visits may be necessary if the signature of pathogenic and protective antibodies is determined simultaneously with serology to confirm diagnosis of RA. Testing patients with early, confirmed RA would represent the most cost‐effective approach, as these individuals benefit most from timely and effective treatment. Extending such testing to individuals at risk of RA would require highly sensitive and specific biomarkers, as well as clear health‐economic justification for preventive interventions. At early RA stage the information about whether a patient has a more pathogenic or protective autoantibody profile, and is therefore prone to a more severe or a milder disease, could be used to guide the treatment decisions for better likelihood for effective treatment. In case the autoantibody signature could predict who would most likely benefit from a certain treatment, or whether a certain treatment will most likely be ineffective in an individual, such test would be meaningful at any stage of RA, or at least when a patient experiences a lack of response to a treatment. Screening asymptomatic or individuals at risk for RA for pathogenic/protective ACPAs may guide in decisions as to whether the benefits of a treatment before RA diagnosis, such as abatacept [125], would outweigh the possible risks in an individual. Furthermore, especially when an individual lacks the classical serological markers RF and anti‐CCP, a positive novel test may provide these individuals an access to preventive treatment [98]. Such markers identifying seronegative at‐risk‐for RA individuals would also allow recruitment of these individuals, comprising up to 30% at the early RA stage, to future clinical trials of preventive medication.

The ability to predict treatment responses in patients with RA remains an unmet need which together with the relatively high unresponsiveness rate to pharmacotherapy delays effective treatment for a large proportion of patients. Presence of ACPA has been indicated to associate with a better treatment response to abatacept, but not to TNF inhibitors [126]. Abatacept modulates T cell effector functions but has not consistently been shown to affect ACPA levels in RA. Nevertheless, some studies found that ACPA levels decreased after abatacept treatment and the conversion to seronegative status during the abatacept treatment has been shown to associate with better radiographic outcomes and sustained remission [127, 128]. These observations raise the possibility that treatment decisions could be guided not only by ACPA but by the balance of protective and pathogenic autoantibody specificities—to identify those most likely benefiting from abatacept. Whether the efficacy of abatacept depends on the patient's individual balance of protective and pathogenic ACPA specificities remains to be investigated. It is conceivable that depletion of protective ACPAs could counteract treatment efficacy, whereas a reduction in predominantly pathogenic ACPAs could enhance therapeutic responses. Such dynamics would open new possibilities for precision medicine.

IVD testing for RF and anti‐CCP is currently centralized and will probably mainly remain due to requirements of analytical accuracy and sensitivity as well as traceability and ease in following the quality control schemes. Advantages of qualitative and semi‐quantitative near‐patient (NPT) and point‐of‐care testing (POCT), a screening tool in primary care or in rural areas, could support earlier and accurate diagnosis of joint inflammatory diseases as an adjunct to centralized serology testing confirming diagnosis. However, more complex testing for individualized signatures, such as screening for pathogenic and protective autoantibodies, is likely to require centralized testing in clinical laboratories with specialized equipment. Serum and plasma are the major sample matrixes with standardized collection methods for IVD testing of autoantibodies, but alternative less invasive sample matrixes such as saliva may be applicable for autoantibody detection in settings where serum collection is less feasible, for example, in pediatric care. The lack of standardized saliva collection methods, limitations in analytical sensitivity, and the limited evidence supporting the scientific validity and analytical performance of saliva, together with commercial uncertainty, may hinder the emergence of saliva‐based IVD testing for autoantibodies.

Overall, the unmet need in RA for better prognostic factors remains, but ACPA fine specificities indicative of future pathogenesis or protection may provide means for earlier and more accurate diagnosis, guidance in choosing an individualized treatment option for those with risk of RA but also for those with early or established RA.

## Funding

This work was supported by Vetenskapsrådet, 2024‐02575; National Key Research and Development Program of China, 2024GH‐ZDXM‐34; National Natural Science Foundation of China, W2431021.

## Conflicts of Interest

R.H. is a founder of the startup company Vacara AB. O.S. is employed and Y.H. is partially employed by Vacara. O.S., Y.H. and R.H. are co‐inventors of Vacara patent application. C.G., E.L. and Z.X. has no confict of interest to declare.

## Data Availability

The data that support the findings of this study are available on request from the corresponding author. The data are not publicly available due to privacy or ethical restrictions.
